# Combatting tobacco industry lobbyists in public health

**DOI:** 10.18332/tid/172140

**Published:** 2023-10-20

**Authors:** James Matheny, Annie Tegen, Meredith Berkman, Nichelle Gray, Benjamin Verhagen, Vela Sree

**Affiliations:** 1 Health Promotion Research Center, Oklahoma City, United States; 2 Independent researcher, Washington, United States; 3 Parents Against Vaping E-cigarettes, New York City, United States; 4 Action on Smoking and Health, Washington, United States

**Keywords:** tobacco industry, lobbyists, laws, public health, corrective statements

Lobbyists can be of tremendous benefit to public health when guided by truth and good intent. Not so when they take their orders from an industry that conspires to commit fraud while marketing addictive and deadly products. In the realm of lobbying activities, Big Tobacco spares no effort in its relentless pursuit to interfere in the vital process of lawmaking. At all levels of government, tobacco industry lobbyists exert extensive and destructive influence.

At the federal level alone, public records indicate that the tobacco industry has registered an astounding 236 lobbyists in 2023^1^. Three out of four (76.3%) are former government employees, an unusually high proportion among industries^2^. These aggressive ‘revolving door’ hiring practices reflect a pattern of interference that compromises public health nationwide.

To monitor state level activity, Action on Smoking and Health (ASH) has published a 2023 online ‘tracker’ of tobacco industry lobbying registrations for all 50 US states and the District of Columbia, including an interactive map allowing comparisons between jurisdictions^3^. It is the second edition of the tracker that was launched in 2021 as the first such resource to use public records from state lobbying registration websites to compile a uniform, user-friendly dataset.

The 2023 ASH tracker identifies a total of 927 state-level tobacco industry lobbying registrations (an average of 18.2 per state), listing 856 tobacco industry lobbyists or lobbying firms (an average of 16.8 per state). The actual number of lobbyists who work on behalf of the tobacco industry is likely even higher. Twelve states -Alabama, Arizona, Arkansas, California, Colorado, Illinois, Indiana, Massachusetts, Michigan, Nebraska, New York, and Pennsylvania- allow lobbying firms to register instead of individual lobbyists^3^. Because most lobbying firms employ multiple lobbyists, the number of individuals within a firm who represent a given client cannot be determined from public records in these states.

Almost two-thirds of the state-level tobacco industry lobbying registrations for 2023 are directly associated with federally adjudicated racketeers^3^. The two largest US tobacco companies, Altria (formerly Philip Morris Companies, Inc.) and Reynolds American, Inc. (RAI), were found in 2006 to have engaged in a five-decade organized conspiracy to commit fraud under the US Racketeer Influenced and Corrupt Organizations (RICO) Act^4^. With 293 registered lobbyists or lobbying firms, Altria maintains a presence in all 50 states and DC. RAI registered a total of 189 lobbyists or firms in 2023, covering 49 states and DC. Juul, which holds an irrevocable licensing arrangement with Altria, registered 95 lobbyists or firms in 2023, covering 39 states and DC. Adding to their combined strength, lobbyists who represent tobacco companies not named in the RICO verdict often work closely with those who are^5^.

Unfortunately, lobbying registrations only tell part of the story. Using public records to monitor tobacco industry lobbying activity is comparable to viewing the tip of an iceberg above the waterline, where the visible portion represents a small portion of the total mass. Tremendous influence is applied beneath the surface, taking place behind closed doors and hidden from public scrutiny^5,6^. Internal documents made public by court order reveal how the tobacco industry builds networks and alliances to more effectively interfere in lawmaking, using funds and favors to cultivate partnerships with front groups, fellow lobbyists, and elected officials^5,7-10^.

Further extending the tobacco industry’s reach, many of its lobbyists and lobbying firms also represent other clients^3,11^. Often, these include organizations with missions at odds with those of the tobacco industry. Groups committed to advancing health, education, or other civic-minded endeavors can prevent inherent conflicts of interest and avoid lending credibility through association by setting internal policies to not hire any lobbyist or firm representing the tobacco industry.

Big Tobacco is best defined by its duplicitous behavior. Under the federal RICO law, prosecutors had to prove that the tobacco companies’ organized conspiracy to commit fraud was likely to continue^4^. As predicted, their lobbyists and allies have never ceased to misinform lawmakers and, by extension, misinform laws^12-14^. Where laws are kept weaker, smoking rates remain higher^15^. Nowhere is their duplicity clearer than when they promote as beneficial self-serving legislation of their own design. The tobacco industry wrote many statutes still in effect today, including state preemption of local laws^5,12-14^. The ever-accumulating harms are incalculable.

The latest development in the federal RICO case against tobacco companies is a court order requiring the posting of ‘corrective’ statements inside retail stores^16^. An estimated 220000 US stores must help disseminate the 17 statements for at least 21 months, from 1 October 2023 to 30 June 2025, as part of their merchandising agreements with tobacco companies. Though unprecedented and important, the corrective statements alone will likely be insufficient to affect any meaningful improvement in public health^17^. Corrective action is essential.

Inside state capitols, dedicated public health lobbyists serve critical roles in educating lawmakers on the policy implications of tobacco industry fraud. The vast experience and institutional knowledge of those who lobby for the American Cancer Society Cancer Action Network, American Heart Association, American Lung Association, Campaign for Tobacco-Free Kids, medical associations, and other groups committed to improving public health are essential for assisting legislative champions who will persistently challenge tobacco industry interference and reinvigorate efforts to enact effective laws in their respective states.

To accomplish their ambitious objectives, public health heroes working inside State Capitols need the active support of coalitions, networks, faith groups, business owners, and individuals communicating directly with their elected officials. While the deep pockets and resources of the tobacco industry are virtually unrivaled, nothing is more powerful than the authentic, combined voices of constituents fighting to create a healthier future for their communities.

Those voices cannot be ignored when amplified and sustained through press events, op-eds, letters to editors, social media and other activities that publicly echo the call to action. The corrective statements posted in stores across the country present newsworthy local, state, and national ‘earned media’ opportunities that will greatly extend their reach and impact. Research shows that raising public awareness of tobacco industry fraud aids smoking cessation, prevents youth initiation, and increases lawmakers’ support for stronger tobacco-related laws^18-20^. There is a positive exposure-response effect to all such efforts^21^ – the more, the better.

Truth is power, but only if told. The tobacco industry lobby is strong, but vulnerable. This critical battle for public health can be won in every state, and there is no better time than now.

## Data Availability

Data sharing is not applicable to this article as no new data were created.
